# Kruppel-like factor 15 is required for the cardiac adaptive response to fasting

**DOI:** 10.1371/journal.pone.0192376

**Published:** 2018-02-06

**Authors:** Keiki Sugi, Paishiun N. Hsieh, Olga Ilkayeva, Shamanthika Shelkay, Bridget Moroney, Palvir Baadh, Browning Haynes, Megan Pophal, Liyan Fan, Christopher B. Newgard, Domenick A. Prosdocimo, Mukesh K. Jain

**Affiliations:** 1 Case Cardiovascular Research Institute and Harrington Heart & Vascular Institute, University Hospitals Case Medical Center and Case Western Reserve University School of Medicine and University Hospitals Case Medical Center, Cleveland, OH, United States of America; 2 Department of Medicine, University Hospitals Case Medical Center and Case Western Reserve University School of Medicine and University Hospitals Case Medical Center, Cleveland, OH, United States of America; 3 Department of Pathology, Case Western Reserve University, Cleveland, OH, United States of America; 4 Sarah W. Stedman Nutrition and Metabolism Center, Departments of Medicine and Pharmacology and Cancer Biology, Duke University School of Medicine, Durham, NC, United States of America; Brigham and Women's Hospital, Harvard Medical School, UNITED STATES

## Abstract

Cardiac metabolism is highly adaptive in response to changes in substrate availability, as occur during fasting. This metabolic flexibility is essential to the maintenance of contractile function and is under the control of a group of select transcriptional regulators, notably the nuclear receptor family of factors member PPARα. However, the diversity of physiologic and pathologic states through which the heart must sustain function suggests the possible existence of additional transcriptional regulators that play a role in matching cardiac metabolism to energetic demand. Here we show that cardiac KLF15 is required for the normal cardiac response to fasting. Specifically, we find that cardiac function is impaired upon fasting in systemic and cardiac specific *Klf15*-null mice. Further, cardiac specific *Klf15*-null mice display a fasting-dependent accumulation of long chain acylcarnitine species along with a decrease in expression of the carnitine translocase *Slc25a20*. Treatment with a diet high in short chain fatty acids relieves the KLF15-dependent long chain acylcarnitine accumulation and impaired cardiac function in response to fasting. Our observations establish KLF15 as a critical mediator of the cardiac adaptive response to fasting through its regulation of myocardial lipid utilization.

## Introduction

The adult heart sustains contractile function regardless of fuel supply, in the face of enormous metabolic demand. Indeed, from an ATP pool of approximately 5 μmol/g wet weight, the heart uses 0.5 μmol/g wet weight per second and therefore perpetually verges on ATP depletion [1]. Although the heart demonstrates a preference for the oxidation of fatty acids, as approximately 70% of the ATP generated in the myocardium is derived from them, ~20% comes from glucose and lactate, and the remainder from diverse substrates (e.g. ketones) [2]. Furthermore, the heart is endowed with the capacity to adapt substrate utilization to match the systemic environment. During fasting, the myocardium increases its dependence on oxidative catabolism of fatty acids and inhibits glycolysis, events which occur simultaneously with a rise in expression of genes involved in fatty acid oxidation [3, 4]. The importance of this adaptive capacity to enhance lipid utilization is illustrated by the clinical features of patients with inborn errors of myocardial fatty acid oxidation, where episodic manifestations of the disease often occur following fasting, presumably as the heart relies more heavily on fatty acid oxidation to meet its energetic demands. [5]

Myocardial lipid utilization is a complex multi-step process [2]. Fatty acids enter the myocardium via passive diffusion or facilitated by carriers including fatty acid translocase (FAT)/CD36, the plasma membrane isoform of fatty acid binding protein (FABPpm), or fatty acid transport protein (FATP) 1/6, with CD36 accounting for 50–60% of fatty acid oxidation [6]. Long chain cytosolic fatty acids are esterified by fatty acyl CoA synthetase (FACS) and, if destined for oxidation, further transferred to carnitine via the mitochondrial enzyme carnitine palmitoyltransferase 1 (CPT1) and shuttled across the outer mitochondrial membrane. Translocation of acylcarnitines across the inner mitochondrial membrane is accomplished by a carnitine:acylcarnitine translocase (Slc25a20), moving the fatty acid moiety into the mitochondrial matrix, and simultaneously generating free carnitine. Carnitine palmitoyltransferase 2 (CPT2) subsequently converts long-chain acylcarnitines into acyl CoA species, which are free to enter the β-oxidation pathway.

Fasting induced alterations in myocardial lipid utilization are achieved, in part, through the activity of several key transcriptional regulators. Work performed by others over the last decade has identified roles for nuclear receptors including the peroxisome proliferator-activated receptors (PPARs), estrogen-related receptors (ERRs), and nuclear respiratory factors 1 and 2 (NRF-1 and -2) in the regulation of genes, among others, involved in cardiac fatty acid oxidation [7]. Recently, we have provided evidence that a zinc-finger DNA-binding transcription factor, Kruppel-like factor 15 (KLF15), is also a critical regulator of myocardial substrate metabolism, especially lipid utilization, and cooperates with PPARα to coordinately regulate gene expression [8, 9]. Further, KLF15 is required for PPARα mediated induction of canonical fasting-inducible gene targets [8, 9].

Given our observations, we therefore postulated that KLF15 regulation of cardiac metabolism might be necessary for metabolic adaptation to substrate availability, as occurs under fasting regimens. Here, we demonstrate that *Klf15*-null hearts exhibit impaired function during fasting, coincident with an accumulation of long chain acyl-carnitines and decrease in transcript levels of *Slc25a20*. These phenotypes are rescued by a diet high in short chain fatty acids, which do not require transport into the mitochondria. Together, our findings suggest KLF15 is critical to the cardiac fasting response and point to a model whereby KLF15 regulation of *Slc25a20* during the fasting state enables transport of long chain acylcarnitine species into mitochondria for oxidation.

## Materials and methods

### Animal models

Animal protocols were approved by the Institutional Animal Care and Use Committee at Case Western Reserve University. Protocols were in accordance with the NIH Guide for the Care and Use of Laboratory Animals. *Klf15-/-* and cardiac-specific KLF15-cKO mice have been previously described [10, 11]. Male, age-matched controls on a C57Bl/6 genetic background were used. Mice were maintained with ad libitum access to water and standard laboratory chow (Laboratory diet P3000; 4.5% fat by weight, 14% kilocalories). Barrier facility had a 12 hour light/dark cycle and was temperature and humidity controlled. For fasting experiments, food was removed at indicated times and lasted for up to 48 hours in duration. Where indicated, mice were fed a short chain fatty acid enriched diet (SCD: Harlan Teklad TD.09849) for 10-weeks as previously described [12].

### Plasma parameters and tissue lipids

Hearts and plasma were rapidly harvested from WT and KO mice fed ad libitum or fasted for two days. Lipids were extracted from ventricles by the Folch method. Cardiac fatty acid and triglyceride content was quantified by thin-layer chromatography by the NIH-MMPC / Lipid Analytic Core at Vanderbilt University. Plasma free-fatty acids and triglycerides were quantified by the Vanderbilt NIH Mouse Metabolic Phenotyping Core. Plasma glucose measurements were performed using the glucose colorimetric / fluorometric assay kit from BioVision according to the manufacturer’s instructions.

### Acylcarnitine measurements

Hearts were rapidly harvested from WT and KO mice fed ad libitum or fasted for two days. Tissue homogenates (50 mg/mL) were prepared using a polytron in 50% acetonitrile and 0.3% formic acid. 45 acylcarnitine species (including short, medium, and long-chain) were analyzed by tandem mass spectrometry (MS/MS) using sample preparation methods described previously [13, 14]. The data were acquired using a Waters triple quadrupole detector equipped with AcquityTM UPLC system and controlled by MassLynx 4.1 software platform (Waters, Milford, MA).

### Echocardiographic analysis

Transthoracic echocardiography was performed in anesthetized mice using the Vevo 770 High Resolution Imaging System (Visual Sonics) and the RMV-707B 30-MHz probe as previously described [10]. Cardiac function was assessed at the mid papillary muscle level in M-mode short axis images.

### RNA extraction and QPCR

Heart tissue samples were disrupted in PureZOL^TM^ (Biorad) in a Tissue-lyzer (Qiagen) using stainless steel beads (30 Hz for a total of 4 min). Aurum^TM^ (Biorad) RNA isolation kit was used to isolate total RNA according to manufacturer’s directions. For qPCR analysis, total RNA was loaded onto columns and DNase treated before being transcribed to complementary DNA using iScript^TM^ (Biorad) according to manufacturer’s directions. TaqMan method was used for qPCR analysis (using the Roche Universal Probe Library System) using an ABI Step One Plus Real-Time PCR System. ΔΔ*Ct* method (with normalization genes indicated in figure legend)was used to assess relative expression. Primer sequence for *Slc25a20*: Forward 5’-ATCCGCGGCTTCTACAAAG-3’, Reverse 5’-TACATCCCACTGGCAGGAAC-3’. Primer sequence for *Cpt1b*: Forward 5’-TGCCTTTACATCGTCTCCAA-3’, Reverse 5’-GGCTCCAGGGTTCAGAAAGT-3’. Primer sequence for *Cpt2*: Forward 5’-CCAAAGAAGCAGCGATGG-3’, Reverse 5’-TAGAGCTCAGGCAGGGTGA-3’. Primer sequence for *Fabp3*: Forward 5’-CTTTGTCGGTACCTGGAAGC-3’, Reverse 5’-TGGTCATGCTAGCCACCTG-3’. Primer sequence for *Glut4*: Forward 5’-TCGTCATTGGCATTCTGGT-3’, Reverse 5’-AGCAGTGGCCACAGGGTA-3’. Primer sequence for *Glut1*: Forward 5’-ATGGATCCCAGCAGCAAG-3’, Reverse 5’-CCAGTGTTATAGCCGAACTGC-3’. Primer sequence for *Ppib*: Forward 5’-TTCTTCATAACCACAGTCAAGACC-3’, Reverse 5’-ACCTCCGTACCACATCCAT-3’.

### Western blot analysis

Protein was extracted and homogenized from heart tissue samples in RIPA buffer (Sigma-Aldrich, R0278) containing proteinase/phosphatase inhibitor cocktail (Roche). Samples in SDS sample buffer were run on SDS-PAGE and immunoblotted with anti-CACT (Sigma, 1:1000) and anti-α-tubulin (Cell Signaling, 1:2000). Secondary HRP-conjugated antibodies were from Cell Signaling and chemiluminescent detection reagent was from Thermo Scientific. Bio-Rad Quantity One software was used for quantitation. Two-tailed Student's *t*-test for unpaired data was used

### Data analysis

All error bars depict SEM, and data is expressed as means. In experiments comparing the means of two normally distributed groups, two-tailed Student's *t*-test for unpaired data was used. In experiments comparing the means of normally distributed groups with multiple treatments, one-way analysis of variance (ANOVA) with the Tukey post hoc test was used. Statistical significance defined as *p* < 0.05.

## Results

### Cardiac KLF15 is required for the heart’s functional adaptation in response to fasting

**Fig 1:**
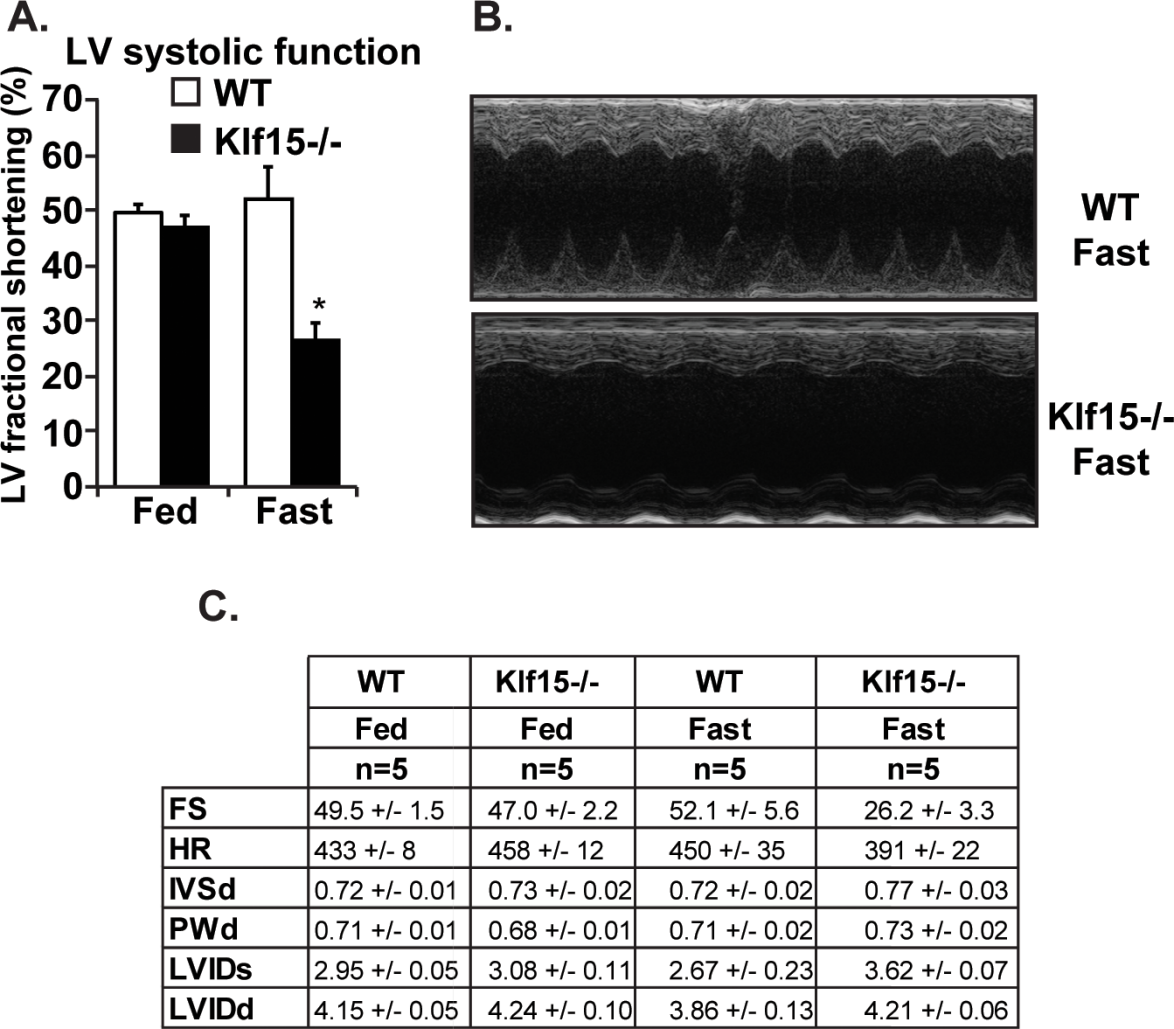
Systemic KLF15 is required for the heart’s functional adaptation in response to fasting. (A) Left ventricular fractional shortening from echocardiography performed in wild-type (WT) vs. systemic *Klf15*-null (*Klf15-/-*) under fed vs. 48 hours fasting conditions, (n = 5), *P,0.05 vs. WT Fast. (B) Representative echocardiography image from WT vs. *Klf15-/-* following a 48 hour fast. (C) Tabular representation of echocardiography data in WT vs. *Klf15-/-* under fed vs. 48 hour fasting conditions.

**Fig 2:**
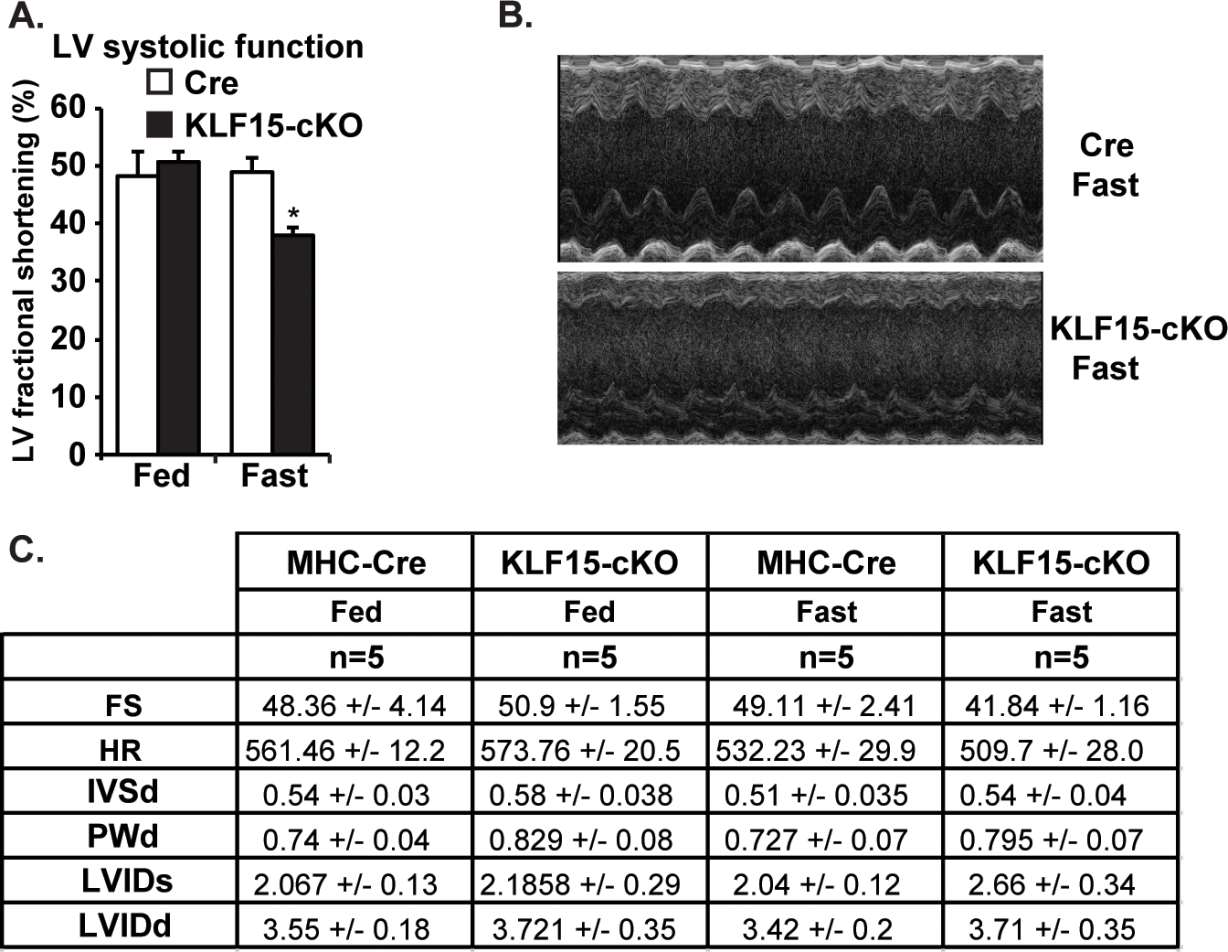
Cardiac KLF15 is required for the heart’s functional adaptation in response to fasting. (A) Left ventricular fractional shortening from echocardiography performed in control (MHC-Cre) vs KLF15-cKO under fed vs. 48 hours fasting conditions, (n = 5), *P<0.05 vs. MHC-Cre Fast. (B) Representative echocardiography image from MHC-Cre vs. KLF15-cKO following a 48 hour fast. (C) Tabular representation of echocardiography data in MHC-Cre vs. KLF15-cKO under fed vs. 48 hour fasting conditions.

Cardiac *KLF15* is highly responsive to diverse physiologic conditions requiring enhanced fatty acid utilization / oxidation, including fasting, postnatal cardiac maturation, and the circadian sleep/wake transition [11, 15, 16]. To gain initial insights into the functional role of KLF15 in controlling lipid metabolism, we reasoned that its absence under fasting conditions might render the heart susceptible to dysfunction. As such, we first utilized systemic *Klf15*-null animals and examined cardiac function under fed and fasted conditions. As shown in Fig 1A & 1B, cardiac function was significantly reduced in the fasted state in the absence of KLF15. This decreased cardiac function occurred in the absence of overt signs of remodeling / hypertrophy (Fig 1C). However, given the numerous metabolic disturbances observed in the systemic *Klf15*-null line following fasting (e.g. alterations in glucose and amino acid levels), we performed a similar set of experiments in the cardiac restricted KLF15 knockout model. As shown in Fig 2A–2C, we observed a similar KLF15-dependecy on cardiac function following fasting suggesting a cardiac specific and cell autonomous role.

### Cardiac specific deletion of KLF15 alters lipid utilization

**Fig 3:**
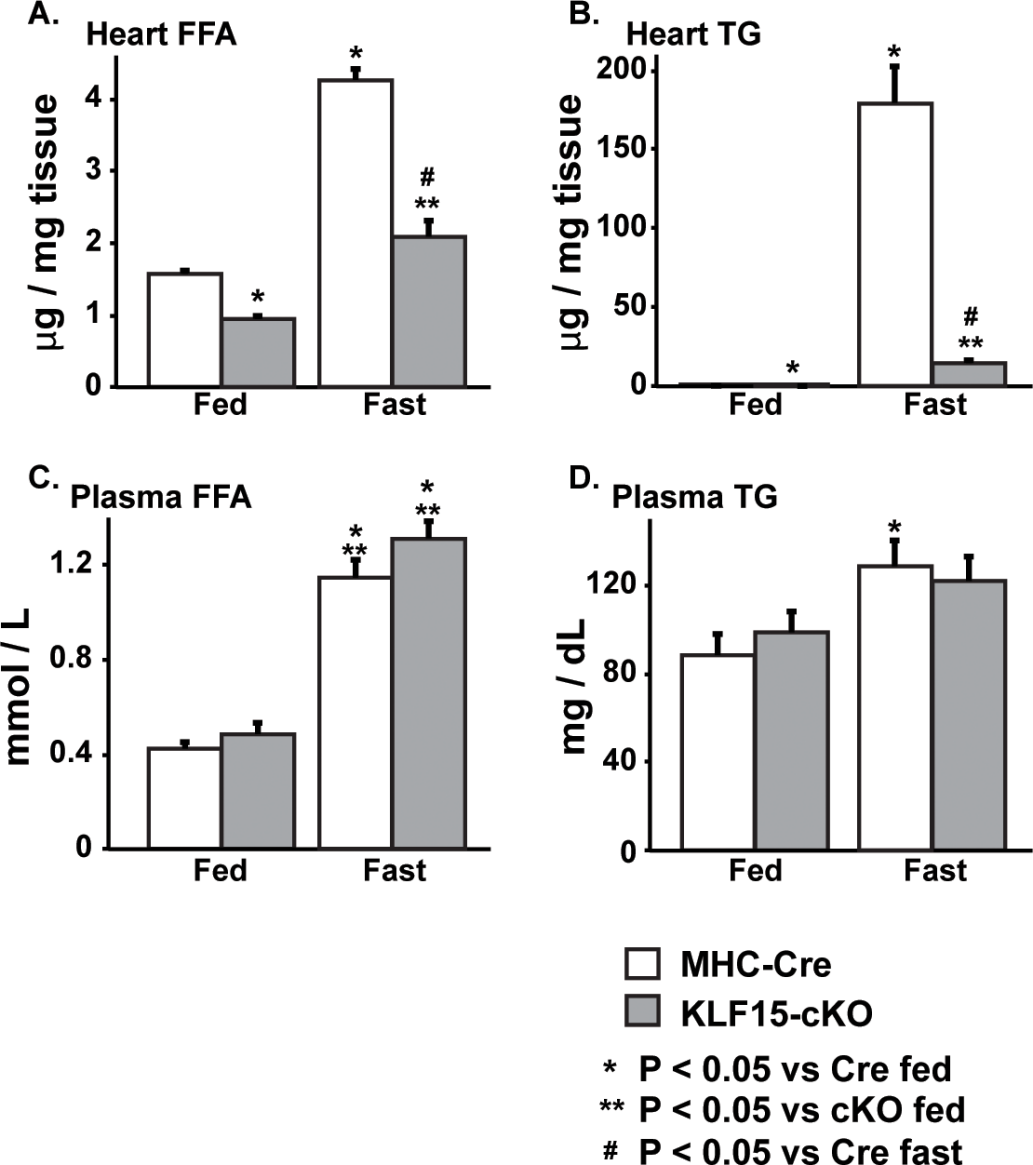
Cardiac specific deletion of KLF15 alters tissue and plasma levels of free fatty acids and triglycerides. Cardiac FFA (A) and TG (B) levels in control (MHC-Cre) vs. KF15-cKO following 48 hours fasting, (n = 5), *P<0.05 vs. Cre Fed, **P<0.05 vs. CKO Fed, # P<0.05 vs. Cre Fast. Plasma FFA (C) and TG (D) levels in control (MHC-Cre) vs. KLF15-cKO following 48 hours fasting, (n = 5), *P<0.05 vs. Cre Fed, **P<0.05 vs. CKO Fed, # P<0.05 vs. Cre Fast.

To further investigate the cardiac specific role of KLF15 in controlling lipid metabolism, cardiac and plasma samples were harvested from both control (MHC-Cre) and KLF15-cKO mice in the fed and fasted state. As shown in Fig 3A & 3B, the accumulation of both free fatty acids (FFA) and triglycerides (TG) following fasting is blunted in the absence of cardiac KLF15 expression. Additionally, while we observed the expected increase in circulating FFA and TG levels following fasting, we did not observe a cardiac KLF15 dependency on circulating FFA and TG following fasting (Fig 3C & 3D). Taken together, these data suggest that cardiac KLF15 controls, at least in part, lipid uptake / utilization in the context of physiologic fasting.

**Fig 4:**
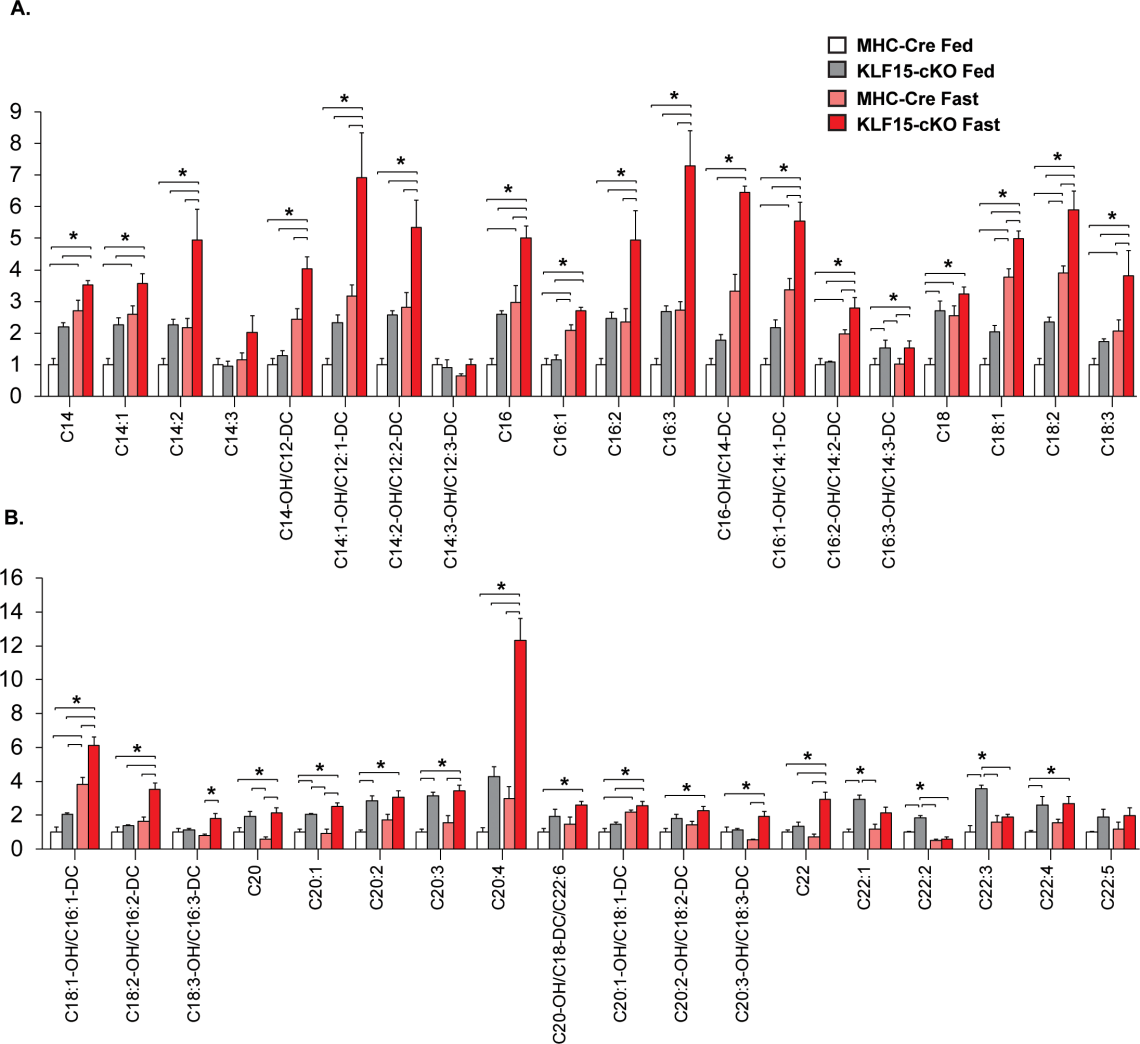
Cardiac specific deletion of KLF15 alters lipid profile. Metabolomic analysis of long chain acylcarnitines in cardiac tissue from control (MHC-Cre) vs. KLF15-cKO with and without 48 hour fast, (n = 5), *P<0.05 by one-way analysis of variance (ANOVA) with the Tukey post hoc test.

To further test this hypothesis, we undertook a metabolomics approach and assessed the cardiac acylcarnitine (AC) profile in both control and KLF15-cKO under fed and fasted conditions. As described previously, AC species (ranging in chain length from 2 to 22 carbons) are formed from their acyl-CoA intermediates by carnitine acyltransferases (CACT; e.g. *Slc25a20*) prior to β-oxidation [17]. These ACs are membrane permeable and can accumulate in both tissue and circulation [18]. Therefore, profiling the AC pool provides a reliable index of lipid flux with even-chain species ranging in length from C6 to C22 representing incomplete utilization of fatty acids [18]. As shown in Fig 4A & 4B, we observed an expected increase in long-chain AC species in MHC-Cre hearts following fasting. Additionally, we found that loss of cardiac KLF15 expression in the fed state gave rise to augmented long-chain AC species, and thus incomplete β-oxidation, an effect that was exacerbated in the fasted state (Fig 4A & 4B). Taken together, these data suggest KLF15 is an important regulator of lipid uptake and utilization in the heart under physiologic states of stress such as fasting.

### Short-chain diet rescues the KLF15-dependent attenuation of cardiac function in response to fasting

**Fig 5:**
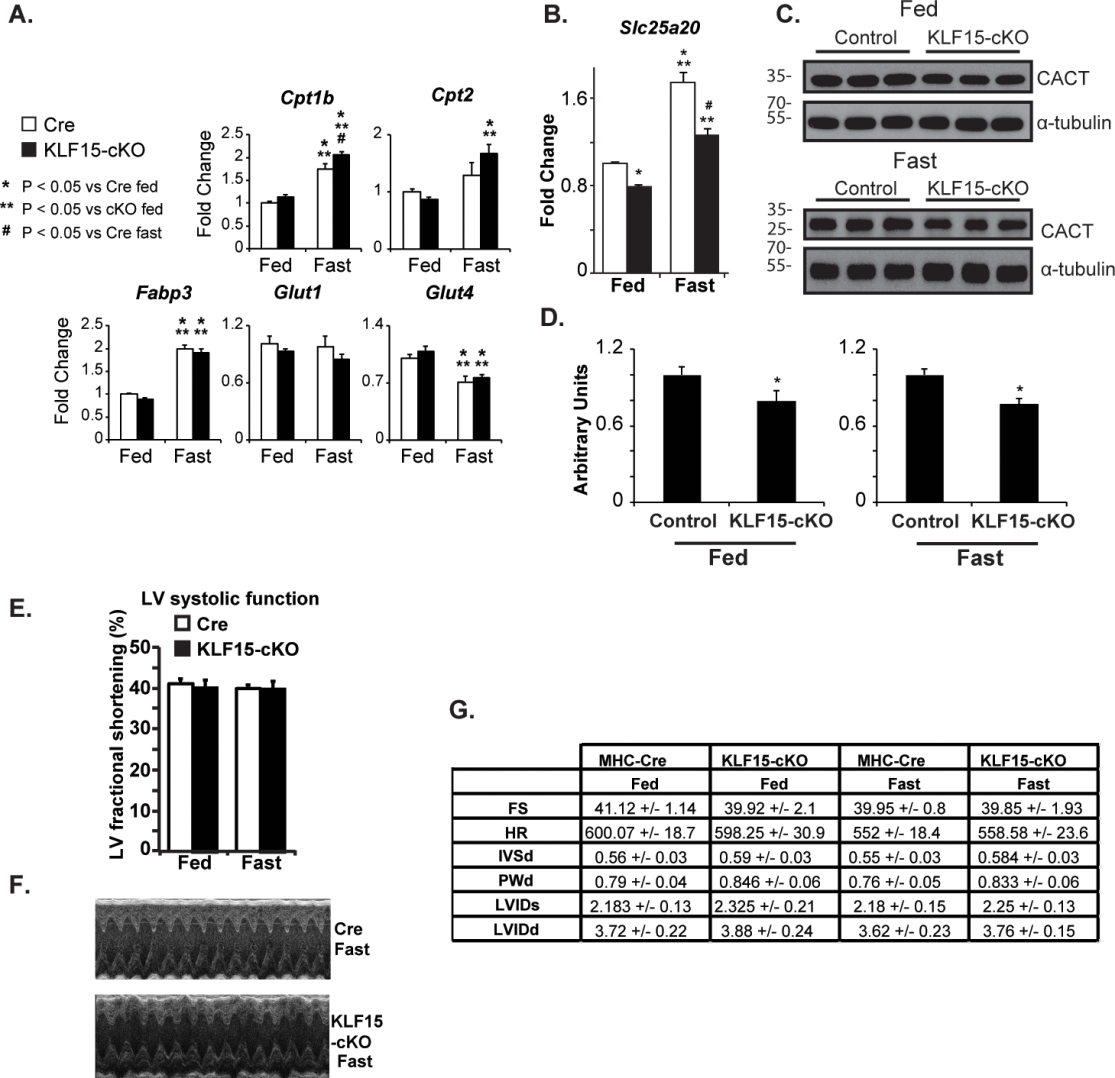
Short-chain diet rescues the KLF15-dependent attenuation of cardiac function in response to fasting. (A) qPCR analysis of expression of transporter genes in MHC-Cre vs. KLF15-cKO under fed vs. 48 hour fasting conditions. *P<0.05 vs. Cre Fed, **P<0.05 vs. CKO Fed, # P<0.05 vs. Cre Fast. Values normalized to *Ppib*. (B) *Slc25a20* expression (qPCR) in MHC-Cre vs. KLF15-cKO under fed vs. 48 hour fasting conditions. *P<0.05 vs. Cre Fed, **P<0.05 vs. CKO Fed, # P<0.05 vs. Cre Fast. Values normalized to *Ppib*. (C) Western blot analysis of CACT levels in MHC-Cre vs KLF15-cKO under fed and 48 hour fasting conditions. α-tubulin used as loading control. (D) Quantification of data in C (n = 3 per group). Two-tailed Student's *t*-test for unpaired data was used. *P<0.05. (E) Left ventricular fractional shortening from echocardiography performed in control (MHC-Cre) vs. KLF15-cKO under fed vs. 48 hours fasting conditions following 10 weeks of short-chain fatty acid diet, (n = 10). (F) Representative echocardiography image from MHC-Cre vs. KLF15-cKO following 48 hours fasting and 10 weeks of short-chain fatty acid diet. (G) Tabular representation of echocardiography data in MHC-Cre vs. KLF15-cKO under fed vs. 48 hour fasting conditions following 10 weeks of short-chain fatty acid diet.

**Fig 6:**
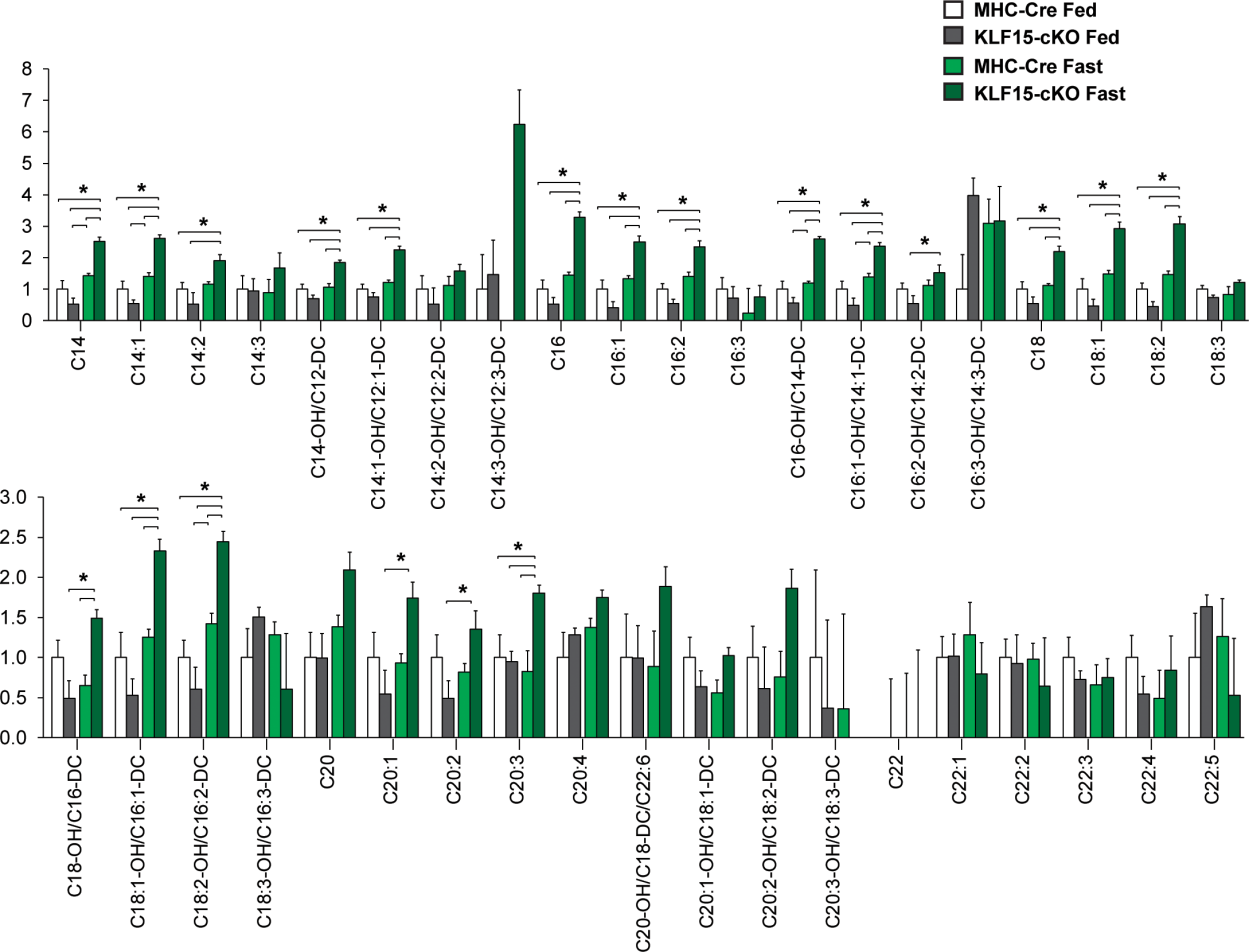
Short-chain diet rescues the KLF15-dependent accumulation of long chain acylcarnitines in response to fasting. Metabolomic analysis of long chain acyl-carnitines in cardiac tissue from control (MHC-Cre) vs. KLF15-cKO with and without 48 hour fast, (n = 6) following 10 weeks of short-chain fatty acid diet, *P<0.05 by one-way analysis of variance (ANOVA) with the Tukey post hoc test.

**Fig 7:**
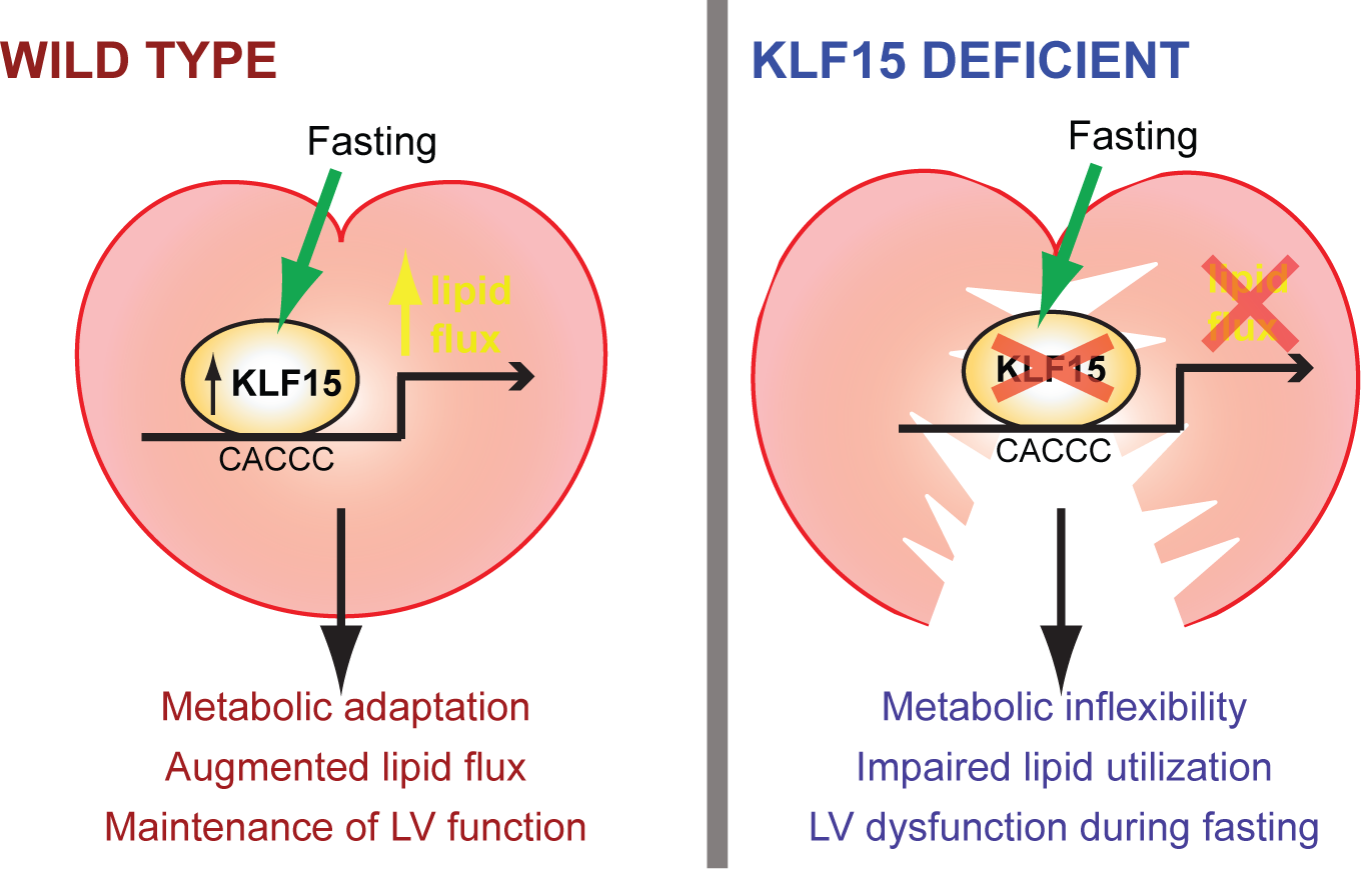
Schematic representing KLF15 as a regulator of the cardiac adaptive response to fasting.

Given the above results, we next sought to rescue the fasting and KLF15 dependent cardiac dysfunction. Towards this end, we fed control and KLF15-cKO mice a diet rich in short-chain fatty acids which bypass the *Slc25a20* transporter and drive β-oxidation. We assessed the expression of *Slc25a20*, whose gene product is the rate-limiting step in converting long-chain acyl-CoAs to the cell permeable acylcarnitine species. As shown in Fig 5A, mRNA transcript levels of other transporters involved in glucose transport and fatty acid transport into mitochondria, are either unaffected or slightly increased by cardiac specific loss of KLF15 under fed or fasted conditions. In contrast, the fasting induction of *Slc25a20* gene expression is dependent on KLF15 (Fig 5B). Levels of CACT, the gene product of *Slc25a20* are also lower (Fig 5C and 5D). Furthermore, following 10 weeks of short-chain diet, cardiac function was assessed in both the fed and fasted states. As shown in Fig 5E–5G, 10 weeks of SCD rescued the KLF15-dependent cardiac dysfunction following fasting. Importantly, we then performed metabolomics to assess AC species, and, as shown in Fig 6, the augmentation of long-chain AC species in the fed state after loss of cardiac KLF15 expression is nearly abolished with 10 weeks of SCD and is strongly suppressed in the fasted state (Table 1). Taken together, these data suggest KLF15 is necessary in the cardiac adaptive response to fasting through a mechanism involving coordinated expression of *Slc25a20*, which drives appropriate AC conversion for β-oxidation (Fig 7).

**Table 1:** Change in long chain acylcarnitine profiles after loss of cardiac KLF15 expression.

Diet	Normal chow	Normal chow	Short chain fatty acid enriched	Short chain fatty acid enriched
Condition	Fed	Fast	Fed	Fast
Genotype comparison	MHC-Cre vs KLF15-cKO			
**Long chain acylcarnitines**				
**C14**	NC	NC	NC	**+**
**C14:1**	NC	NC	NC	**+**
**C14:2**	NC	**++**	NC	NC
**C14:3**	NC	NC	NC	NC
**C14-OH/C12-DC**	NC	**+**	NC	**+**
**C14:1-OH/C12:1-DC**	NC	**++**	NC	**+**
**C14:2-OH/C12:2-DC**	NC	**+**	NC	NC
**C14:3-OH/C12:3-DC**	NC	NC	NC	NC
**C16**	NC	**+**	NC	**++**
**C16:1**	NC	NC	NC	**+**
**C16:2**	NC	**++**	NC	**+**
**C16:3**	NC	**++**	NC	NC
**C16-OH/C14-DC**	NC	NC	NC	**+**
**C16:1-OH/C14:1-DC**	NC	**+**	NC	**+**
**C16:2-OH/C14:2-DC**	NC	**+**	NC	NC
**C16:3-OH/C14:3-DC**	**+**	**+**	NC	NC
**C18**	**++**	NC	NC	**+**
**C18:1**	NC	**+**	NC	**+**
**C18:2**	NC	**+**	NC	**++**
**C18:3**	NC	**+**	NC	NC
**C18:1-OH/C16:1-DC**	NC	**+**	NC	**+**
**C18:2-OH/C16:2-DC**	NC	**++**	NC	**+**
**C18:3-OH/C16:3-DC**	NC	**++**	NC	NC
**C20**	NC	**++**	NC	NC
**C20:1**	**++**	**++**	NC	NC
**C20:2**	**++**	NC	NC	NC
**C20:3**	**++**	**++**	NC	**++**
**C20:4**	NC	**++**	NC	NC
**C20-OH/C18-DC/C22:6**	NC	NC	NC	NC
**C20:1-OH/C18:1-DC**	NC	NC	NC	NC
**C20:2-OH/C18:2-DC**	NC	NC	NC	NC
**C20:3-OH/C18:3-DC**	NC	**++**	NC	NC
**C22**	NC	**++**	NC	NC
**C22:1**	NC	NC	NC	NC
**C22:2**	**+**	NC	NC	NC
**C22:3**	**++**	NC	NC	NC
**C22:4**	**++**	NC	NC	NC
**C22:5**	NC	NC	NC	NC

Changes in all measured long chain acylcarnitine species after loss of KLF15 expression in fed and fasted conditions, with and without SCD diet. No statistically significant difference between MHC-Cre and KLF15-cKO is represented by (NC), a less than 2 fold increase is represented by (+) and a more than 2 fold increase is represented by (++).

## Discussion

Cardiac metabolic plasticity during fasting, namely the augmentation of lipid utilization, is crucial for its function [2, 19]. Transcriptional regulators coordinate this augmentation, and we establish here that KLF15 is critically required for cardiac lipid flux and function during fasting. In systemic and cardiac specific *Klf15*-null mice, we observe decreased fractional shortening under fasting conditions. Additionally, metabolomics analysis reveals an accumulation of long chain acylcarnitine species in hearts of cardiac specific *Klf15*-null mice during fasting, suggesting an inability of these hearts to adequately manage lipid flux during a nutrient deprived state. This theory is bolstered by our finding that a diet rich in short chain fatty acids, which cross easily into the mitochondria and therefore can act as an alternative energy source, can rescue both the long chain acyl-carnitine abnormalities as well as cardiac fractional shortening. Finally, we find by qPCR analysis that transcript levels of a key transporter of acyl-carnitines, *Slc25a20*, are lowered in cardiac specific *Klf15*-null mice hearts. Together with prior work, these results implicate cardiac KLF15 in the fasting response through its regulation of lipid flux and illustrate the significance of transcriptional control of cardiac metabolism in governing cardiac function.

While coordinated alterations in cardiac metabolism are critical to the normal fasting response, metabolic derangements have also been shown to contribute to various disease states. In diabetic hearts, glucose oxidation is inhibited and there is decreased activation of PI3K-Akt signaling in response to insulin, while fatty acid oxidation is enhanced through increased circulating levels of free fatty acids as well as the aberrant action of PPARα, changes contributing to the development of a diabetes associated cardiomyopathy [20, 21]. Interestingly, the overexpression of PPARα in cardiomyocytes alone is sufficient to phenocopy many of the features of this cardiomyopathy, while PPARα deficiency is protective against a streptozotocin-induced model of diabetic cardiomyopathy [22, 23]. Given our prior description of a KLF15-PPARα molecular module regulating fatty acid oxidation, KLF15 may also play a role in diabetes-induced cardiac dysfunction [9]. Additionally, alterations in substrate usage and their attendant consequences have also been noted in the failing heart, namely a relative, although not absolute, increase in glucose oxidation and decrease in fatty acid oxidation, particularly in late stages of disease [24–28]. Notably, although fatty acid oxidation is reduced, lipid uptake into the cytosol is in fact enhanced, resulting in an accumulation of triglycerides in the cardiomyocyte as well as shunting of lipids into maladaptive non-oxidative pathways, broadly termed lipotoxicity [29, 30]. Previous work from our group has shown that mRNA transcripts of cardiac KLF15 are lowered in rodent and human samples of heart failure, and that the mouse with global loss of KLF15 is more susceptible to heart failure following pressure overload, suggesting a role for KLF15 regulation of fatty acid oxidation in the context of heart failure [8, 16, 31, 32].

The selective accumulation of unoxidized long chain acylcarnitines in the cardiac KLF15 knockout mouse, its exacerbation upon fasting, and its rescue with a diet of short chain fatty acids is reminiscent of the clinical phenotype of patients affected by genetic defects in long chain fatty acid oxidation pathways. These patients, who can harbor mutations in genes of fatty acid transport across the mitochondrial membrane or in specific enzymes of the β-oxidation pathway, exhibit metabolic decompensation during fasting which is alleviated with a diet enriched in medium chain fatty acids and depleted in long chain fatty acids [33–37]. The cardiac sequelae of an inborn error of fatty acid oxidation have been theoretically attributed to toxicity of the long chain acylcarnitine, either through the generation of reactive oxygen species, or through incorporation in the sarcolemma leading to generation of arrhythmias due to its amphiphilic nature; these mechanisms may also be operative in the KLF15 deficient mouse [38]. Indeed, in the diabetic cardiomyopathy model mouse overexpressing PPARα in the heart, Finck et al. observe an accumulation of triacylglycerides containing long chain fatty acids which is rescued upon treatment with a medium chain fatty acid diet [23]. Since, in our model, the relief of long chain acylcarnitine burden occurs simultaneously with the return of normal cardiac function, it seems likely that this accumulation of long chain acylcarnitines is also key to the deterioration in cardiac function of KLF15 knockout mice. Additionally, it remains unclear what mechanisms mediate the decrease in long chain acylcarnitine accumulation in the presence of an abundant alternative lipid energy source such as short chain fatty acids. However, as diets rich in short to medium chain fatty acids have been shown to be useful in some diseases of inborn defects of fatty acid oxidation, it will be valuable to consider their therapeutic value in other diseases in which fatty acid oxidation pathways are dysregulated, including diabetes and advanced heart failure.
